# Observation tool to measure patient-centered behaviors on rounds in an academic medical center

**DOI:** 10.1080/10872981.2021.2024115

**Published:** 2022-01-07

**Authors:** Michelle Sharp, Nicole Williams, Sean Tackett, Laura A. Hanyok, Colleen Christmas, Cynthia S. Rand, Roy C. Ziegelstein, Janet D. Record

**Affiliations:** a Department of Medicine, Johns Hopkins University School of Medicine, Baltimore, Maryland, USA; b Biostatistics, Epidemiology, and Data Management Core, Johns Hopkins School of Medicine, Baltimore, Maryland, USA

**Keywords:** Patient-centered care, patient-centered communication, rounds, shared-decision making, education

## Abstract

**Objective:**

As part of a quality improvement project, we developed and employed an observation checklist to measure patient-centered behaviors during daily rounds to assess the frequency of patient-centered behaviors among a patient-centered care (PCC) team and standard team (ST) rounds.

**Patients and Methods:**

On four general medicine service (GMS) teaching teams at an urban academic medical center in which housestaff rotate, we utilized an observation checklist to assess the occurrence of eight behaviors on inpatient daily rounds. The checklist covered domains of patient-centered communication, etiquette-based behaviors, and shared decision-making. One GMS team is guided by a PCC curriculum that emphasizes patient-centered communication strategies, but not specifically behaviors during bedside rounds.

**Results:**

Between August 2018 and May 2019 a trained observer completed 448 observations of patient rounding encounters using the checklist. Across all teams, 46.0% of the 8 behaviors were performed when possible, with more done on the PCC team (58.0%) than ST (42.0%), p < 0.01.

**Conclusions:**

Performance of patient-centered behaviors during daily rounds was low overall. Despite having no specific instruction on daily rounds, patient-centered behaviors were more frequent among the teams which were part of a PCC curriculum. However, the frequency of observed behaviors was modest, suggesting that more explicit efforts to change rounding behaviors are needed. Our observational checklist may be a tool to assist in future interventions to improve patient-centered behaviors on daily rounds.

## Introduction

Patient-centered care (PCC) involves a partnership between the patient and health care team in which patients participate actively and a relationship is built on mutual trust, empathy, and shared knowledge[1]. Patient-centered communication, an aspect of PCC, is associated with improved patient outcomes, including understanding of assessments and recommendations, adherence to therapy, and patient satisfaction with care [2–6]. Daily rounds are a primary opportunity for patients and their care teams to communicate, and for learners to observe and refine bedside communication skills. However, even when teams are at the bedside there are abundant missed opportunities to explore the patient’s understanding and perspective [7,8], which is important to shared decision-making and effective transitions of care [8–10]. Additionally, despite broad recognition that patient-centered communication is essential for patient care [11–13], studies continue to demonstrate a gap in communication [14–16] and that physicians underestimate their shortcomings in communication [2].

We developed a specific curriculum to teach PCC that is used on one of four general medicine inpatient teaching teams at our medical center [17]. This structured PCC curriculum aims to teach trainees to view patients as individuals and to consider the context of their lives outside the hospital through use of a restructured history and physical exam template, requirements to contact outpatient clinicians, introduction of a structure for exploring medication adherence, and requirements to call all and visit select patients after discharge. Implementation of these curricular activities occurs largely through integration into work done outside the setting of daily rounds, during activities such as gathering initial and follow-up history from patients and collateral sources; talking with patients outside of rounds to discuss patterns of and barriers to medication adherence and to explore their needs and concerns about the transition out of the hospital; and speaking with patients and families after a hospital stay to learn how the transition went and how to improve planning for future patient care transitions. The PCC curriculum has been shown to improve patients’ ratings of physician communication [18] and reduce heart failure readmission rates [19]. Residents have reported improved skills in addressing adherence issues and communicating with patients about the transition out of the hospital [18], and residency program graduates have reported the PCC curriculum had durable impact on their approach to practice [20]. The curriculum does not explicitly provide scripting or guidance on daily rounds, but the curriculum includes quarterly faculty development sessions which have included discussions on conducting daily rounds including prioritizing rounding at the bedside.

To provide baseline information for quality improvement for all teaching teams on our general medicine services, and to assess whether current implementation of the PCC curriculum is associated with performance of fundamental, general patient-centered behaviors on bedside rounds, we developed an observation checklist to assess basic patient-centered behaviors during bedside rounding. Based on literature review of patient-centered care [5,21,22] and consultation with both local and international experts, the checklist addresses three domains: patient-centered communication, etiquette-based behaviors, and shared decision-making. The items on the observation checklist do not map to specific elements in the PCC curriculum, but are fundamental behaviors that are applicable to respectful interactions any patient care environment. In this investigation assessing for an indirect association of the PCC curriculum with behaviors during daily rounds, we hypothesized that more patient-centered behaviors would occur during bedside rounds on the team utilizing the PCC curriculum than other inpatient medicine teams.

## Methods

### Procedures

We conducted an observational, cross-sectional study on the general medical service at Johns Hopkins Bayview Medical Center, a 335-bed urban academic medical center in Baltimore, Maryland with an internal medicine residency program with 59 housestaff. During 82 patient care rounds attended by multiple observers, the checklist was piloted and refined to enhance clarity. Results of the pilot observations are not included here. The final checklist contains eight observable behaviors in three domains: 1) patient-centered communication: a) discussing the plan for the day with the patient, b) primary provider speaking at eye level with the patient, c) incorporation of social touch; 2) etiquette-based behaviors: a) knocking upon entering the patient’s room, b) asking permission to adjust items in the room; c) asking permission to examine the patient; and 3) shared decision-making: a) asking if the patient/family had any questions, and b) noting whether the patient expressed their perspective, hopes, goals, worries, or concerns (Supplemental Figure). The study was reviewed by the Johns Hopkins University Institutional Review Board (IRB00148234) and determined to be exempt.

A single observer (NW), a research coordinator hired for the project, was trained by the authors over several observations prior to the initiation of the study. NW collected data on daily rounds using the checklist to document behaviors on weekdays from August 2018 to May 2019. The observer followed a different team each day for bedside rounds from 8:30 to 11:45 a.m. Generally, each team was observed on the same weekday; each team follows a 4-day cycle of alternating admitting and non-admitting days. Teams were not aware of the specific behaviors on the checklist, but were aware they were being observed to understand what happens during bedside rounds. Given the observational nature of our real-world study setting, it was unfeasible to blind the observer. Patient-team encounters were not observed if there were droplet or airborne isolation precautions, when patients requested the observer not be present, or when the team felt observation might not be appropriate given the patient’s circumstance. In the few instances when completion of a behavior was unclear, ‘not sure’ was recorded. If the item was not relevant (e.g., if the team did not adjust the room) or the item could not reasonably be performed, ‘not applicable’ was documented. The observer recorded time spent outside the room discussing each patient’s care and time spent at the bedside with each patient.

To establish inter-rater reliability, a second observer (MS), who led the pilot and development of the checklist joined on 10 days and independently recorded performance of checklist behaviors.

### Teams observed

The general medical service has a hospitalist service and four housestaff teams. Each housestaff team has an attending physician, a second- or third-year resident, and two interns. A variable number of medical students on clerkships or sub-internships from Johns Hopkins University School of Medicine are also part of the housestaff teams. One of the four housestaff teams uses the PCC curriculum and admits nine patients every 4-day cycle, while each of the other teams admits 10. Team census is typically 7–14 patients for all teams. Residents rotate on the PCC team for 2 to 4 weeks as interns, and again as second- or third-year residents. A few PCC team attending physicians also serve as attendings on standard teams, such that about 10% of the time, a standard team is staffed by an attending familiar with the PCC curriculum. While some trainees on standard teams had been exposed to the PCC curriculum and some standard teams may include an attending who also serves on the PCC team, the standard teams do not utilize the PCC curriculum tools and do not emphasize or require completion of the curricular activities specific to the PCC team. Neither the PCC nor the standard teams were shown the checklist prior to the observations.

### Statistical analysis

Descriptive statistics were tabulated with parametric and non-parametric tests applied as appropriate. ‘Not sure’ and ‘not applicable’ responses were aggregated. For comparisons between the PCC and other teams, data from the other teams were aggregated and the frequency of performance of behaviors was calculated excluding ‘not applicable’ instances. Inter-rater reliability was calculated as percent agreement and kappas. Kappa was artificially low due to the high frequency of performance, so percent agreement is presented here. All analyses were performed using Stata Version 13 (StataCorp LP, College Station, Texas).

## Results

The observer joined rounds for 85 days and was on the PCC team for 24 days (28.2%).

Absolute agreement on checklist items in the 48 encounters where there was a second observer ranged from 79% to 100%.

**Figure 1.**
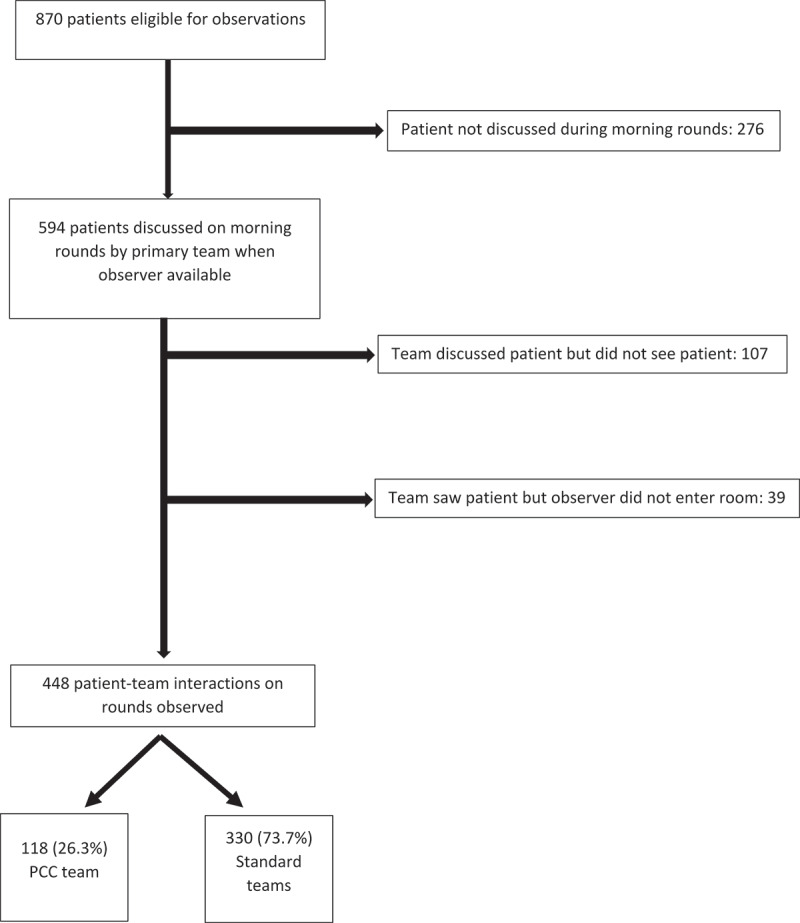
Patient encounters in study PCC, patient-centered care.

Not all patient-team encounters during the eligible time period were included in the study due to various reasons. The reasons patient-team encounters were not included in the study are shown in Figure 1. Teams had a total of 870 patients on their services, however 276 were not discussed during morning rounds. For 107 patients, the team discussed the plan of care but did not see the patient on rounds. The observer did not record observations for 39 patient encounters due to droplet or airborne isolation, or due to a patient or team request (Figure 1). Thus, of the 594 possible observations, 448 (75.4%) were directly observed and had behaviors recorded; of these, 118 (26.3%) were from the PCC team and 330 (73.7%) from other teams.

Characteristics of rounds are shown in Table 1. The average team census was not significantly different between teams (10.0, SD 2.7 on PCC team, vs. 10.2, SD 2.3 on other teams, p = 0.75). The average time spent discussing each patient was not different between teams (PCC team 18.1 minutes, SD 10.5, vs. other teams 18.3 minutes, SD 11.9, p = 0.73). Of the time spent discussing each patient, the PCC team spent more time on average at the bedside (8.5 minutes, SD 4.8, vs. 7.1 minutes, SD 5.2, p < 0.01). For new patient encounters, the proportion of time teams spent at the bedside was not different between the PCC team and other teams (41.4%, SD 19.0 vs. 35.2%, SD 20.4, p = 0.11). The PCC team spent a greater proportion of their rounding time on average at patients’ bedsides on follow-up rounding encounters (58.8%, SD 23.0 vs. 46.8%, SD 21.7, p < 0.01).

**Table 1.** Characteristics of rounds by team PCC, patient-centered care

	PCC team observations N = 118	Standard team observations N = 330	P value (PCC vs standard team)
Mean team census	10.0 (2.7)	10.2 (2.3)	0.75
Patients seen by medical team on morning rounds when observer available	5.6 (2.0)	6.1 (1.8)	0.31
All patient discussions			
Total time discussing each patient,* minutes	18.1 (10.5)	18.3 (11.9)	0.73
Time at patient’s bedside,** minutes	8.5 (4.8)	7.1 (5.2)	<0.01
Time at patient’s bedside** as percentage of total time discussing patient	53.0% (23%)	43.0% (22%)	<0.01
New patient discussions			
Total time discussing each patient,* minutes	27.9 (10.2)	31.3 (11.1)	0.09
Time at patient’s bedside,** minutes	10.9 (5.2)	10.7 (6.7)	0.43
Time at patient’s bedside** as percentage of total time discussing patient	41.4% (19.0%)	35.2% (20.4%)	0.11
Follow-up discussions			
Total time discussing each patient,* minutes	13.4 (6.8)	12.4 (6.2)	0.37
Time at patient’s bedside,** minutes	7.3 (4.1)	5.4 (3.3)	<0.01
Time at patient’s bedside** as percentage of total time discussing patient	58.8% (23.0%)	46.8% (21.7%)	<0.01

^ǂ^Mean census calculated these values based on 77 observation dates, due to missing data on some dates.

*Time discussing patient in any location, including hallway, team room, at bedside, or any other location during rounds.

**Subset of total time discussing each patient, limited to time at the patient’s bedside.

Values are expressed as mean (SD).

Performance of patient-centered rounding behaviors is shown in Table 2. Across all teams, 46.0% of the 8 behaviors were performed when possible, with more done on the PCC team (58.0%) than other teams (42.0%), p < 0.01. The PCC team performed significantly more patient-centered behaviors on daily rounds including discussing the plan for the day with the patient, sitting at eye level when speaking, knocking upon entering, requesting permission to examine the patient, asking the patient if they had any questions, and having the patient’s perspective, hopes, goals, worries, or concerns expressed. There was no significant difference between teams on incorporating social touch and requesting permission to adjust the room.

**Table 2.** Performance of patient-centered behaviors by team PCC, patient-centered care values are expressed as N (% frequency)

	PCC team N = 118*	Standard teams N = 330*	P value (PCC vs standard team)
*Patient-centered communication*			
Plan for day discussed with patient	112 (94.9)	283 (86.8)	0.02
Provider eye level when speaking	50 (44.3)	86 (26.9)	<0.01
Incorporated social touch	63 (53.9)	142 (43.6)	0.06
*Etiquette-based behaviors*			
Knocking upon entering room	81 (78.6)	118 (38.4)	<0.01
Permission to adjust room	14 (28.6)	19 (17.8)	0.13
Permission to examine patient	50 (73.5)	147 (62.6)	0.01
*Shared decision-making*			
Patient asked if he/she had any questions	56 (49.6)	109 (34.3)	<0.01
Patient’s perspective, hopes, goals, worries, or concerns expressed	43 (36.8)	83 (25.6)	0.02

*Not all checklist items were applicable to each patient-team encounter; therefore, the denominator used to calculate rate of performance of each checklist item reflects removal of ‘not applicable’ patient-team encounters for that item.

## Discussion

Using a novel observation checklist for daily rounding to assess performance of fundamental patient-centered behaviors on general medicine teams-, we observed 448 patient-team encounters. The overall performance of patient-centered rounding behaviors across teams was 46%, with the PCC teams performing more of the patient-centered behaviors compared to the other teams, even though the PCC curriculum did not provide specific guidance on including PCC behaviors in daily rounds. PCC teams completed more patient-centered behaviors while spending equivalent time discussing each patient compared to other teams; PCC teams spent a greater proportion of rounding time at the bedside.

In our study, the plan for the day was the item completed most frequently on the checklist (completed overall 88% of the time). Our findings are similar to Real et al. who observed general medicine services and found that 91–96% of the time a general plan review occurred with the patient, which was a part of their intervention protocol [23]. It is also important to note that patient understanding of the hospital plan has also been associated with patient satisfaction [24]. Our study found that while discussing the medical plan for the day was frequently done, there are still more than ten percent of patients who were not informed of the plan.

Sitting at the patient’s bedside has been associated with patients rating their physicians more highly in listening carefully and explaining clearly, but does not take more time [25]. In our study, sitting at eye level occurred more frequently on the PCC team, but still occurred less than half the time. While sitting is not explicitly taught, our PCC curriculum teaches patient-centered communication strategies including use of active listening; exploring patients’ understanding and perspectives on their health, medical conditions, and priorities; and following up on emotional cues, all of which trainees may find are facilitated by sitting at eye level. Our observations suggest that participation in the PCC curriculum, even without specific guidance on rounding practices, might be associated with teams performing more patient-centered communication behaviors, yet with ample room for improvement still. More dedicated teaching around the content and approach to communication on daily rounds may be required to achieve more consistent patient-centered communication during rounds.

Etiquette-based medicine, which has been defined as treating the patient courteously [22] and having respect for the patient’s space and perspective, has been increasingly recognized as important to patients [26–28]. Teams knocked upon entering the patient’s room 78.6% of the time on the PCC team vs. 38.4% of the time on other teams. The latter frequency is similar to that found by Tackett et al. in which knocking occurred 40% of the time among hospitalists [9]. Teams asked permission before beginning the examination around 65% of the time, which was more frequently than observed in Real et al. [23]. Our PCC curriculum prioritizes teaching trainees to seek to understand each patient as an individual and to focus on the humanity and autonomy of each patient. Knocking on a patient’s door, asking permission before adjusting lighting or objects in the patient’s room, and asking permission before beginning the examination on rounds are examples of specific behaviors that can convey respect for a patient’s autonomy and humanity. While many of our faculty are familiar with the concept of etiquette-based medicine, no specific guidance on implementing etiquette-based rounding exists within our curriculum. Our results suggest that participation in the PCC curriculum might be associated with teams considering the patient’s space and perspective more often during rounds. However, if the goal is more frequent etiquette-based behaviors during rounds, more explicit teaching of etiquette-based behaviors may be necessary.

When done well, shared decision-making has been shown to improve patients’ satisfaction, adherence, and overall knowledge of their medical conditions, and the relationship between the provider and patient [29–36]. The PCC curriculum teaches shared decision-making and the importance of using empathic and non-judgmental approaches to explore barriers to adherence and checking a patient’s understanding and comfort with treatment plans. A conversation guide for exploring medication adherence [17], and prompts in a restructured History and Physical admission note, are concrete tools in the PCC curriculum which support shared decision-making around medication management. The curriculum does not explicitly teach seeking the patient’s perspective during rounds. Our checklist assessed shared decision-making in several ways, but the most basic assessment was whether teams asked patients if they had any questions. While this occurred more often on the PCC team than on other teams, it still only occurred in about half of all encounters even on the PCC team. This frequency is similar to other studies [23]. In a descriptive study of baseline communication patterns on rounds, one group found that patients contributed actively to decision-making in 18% of rounds encounters [7]. We sought to measure patient’s active contribution to decision-making through assessing whether the patient’s perspective, hopes, goals, worries, or concerns were expressed. We found that patients voiced their perspectives more frequently to the PCC team during rounds (36.8 vs. 25.6%, p = 0.02), however overall at a very low rate, consistent with other studies [7]. Being able to ask questions as a patient can positively impact patient’s report of communication with physicians [37]. More focus on encouraging questions and a dialogue with patients on rounds in the PCC curriculum may be necessary to improve shared-decision making.

In self-assessment of communication skills and etiquette-based behaviors, physicians often over-estimate their skills and frequency of behaviors [9,28,38], highlighting the importance of an objective instrument to assess behaviors. Indeed, when we showed our results to faculty they were very surprised at the low rates of all behaviors. Our observation checklist may be useful in other settings to evaluate the occurrence of patient-centered behaviors on rounds and assess the effectiveness of quality improvement initiatives.

Our results did not show any significant differences between the census between the two teams. Additionally, we found that the PCC teams and the ST spent a similar amount of time discussing each patient on rounds, but the PCC teams spent significantly more of their time at the bedside with patients (8.5 minutes) compared to ST (7.1 minutes). This difference was most prominent for follow-up presentations, rather than new patient presentations. This modest shift of rounding time to time the bedside may be an indirect response to implementation of the PCC curriculum; if a team has been learning more about each patient’s life context and perspectives throughout a hospital stay, teams may have chosen to use more time at the bedside to engage in shared decision making and tailoring care plans to fit the patient’s life context and priorities. However, teams spent a substantial proportion of time discussing patients away from the bedside, instead using the hallway or team work room. Several studies have found that patients favor a model of rounds conducted primarily at the bedside, specifically finding that patients and families perceive clearer communication from physicians, report enhanced understanding of their medical conditions, and perceive a greater amount of time spent with the physician team with bedside rounds [39–41]. The time spent on rounds with patients in our study is similar to other direct observation studies in which 7 minutes was the average time spent in patient’s rooms [23].

Our study has several limitations. First, our study is a cross-sectional study of rounds at a single center. Further assessment of the observation tool in other settings would be of value. Additionally, our study assessed for an association between participation in a PCC curriculum and performance of fundamental patient-centered behaviors and therefore we did not collect data on patient characteristics. This prevents us from determining if patient-level characteristics such as dementia or delirium contributed to a difference in frequency of the behaviors. We were unable to explore any associations between our findings and patient perspectives on their care or other patient outcomes. In addition, approximately 10% of the PCC attendings also attend on the other general medicine teams and there may be some overlap of the PCC teaching. However, despite the possible overlap, our results still demonstrated a significant difference between the teams. Additionally, it was not possible for our observer to be blinded to team status as PCC vs. other teams because of the different locations of the teams within the hospital.

## Conclusions

Using a piloted and refined observation checklist tool, we found performance of fundamental, broadly applicable patient-centered behaviors during inpatient team rounds was more frequent on a team with a PCC curriculum than on standard teaching teams. However, the frequency of observed behaviors was modest, suggesting that more explicit efforts to change rounding behaviors are needed on all teams. Providing clinicians with results of the observational checklist can provide clinicians with objective feedback on patient-centeredness of rounding behaviors, and this feedback, coupled with efforts to promote being fully present with patients [42] may promote more patient-centered communication and care. Our checklist is not specific to our PCC curriculum and is generalizable to other settings, as it was developed to assess general patient-centered behaviors on daily rounds. The checklist tool may be useful to guide other efforts to evaluate and improve PCC during daily rounds.

JDR had full access to all of the data in the study and takes responsibility for the integrity of the data and the accuracy of the data analysis, including any adverse effects. MS, NW, ST, LAH, CC, CSR, RCZ, and JDR substantially contributed to the study design, data analysis and interpretation, and the writing of the manuscript.

## Supplementary Material

Supplemental Material
