# Supplementary material for: Observation tool to measure patient-centered behaviors on rounds in an academic medical center
Source: Med Educ Online. 2022 Jan 7;27(1):2024115. doi: 10.1080/10872981.2021.2024115 (PMC8745350; doi:10.1080/10872981.2021.2024115)

Figure 1. Patient Encounters in Study

870 patients eligible for observations

Patient not discussed during morning rounds: 276

594 patients discussed on morning rounds by primary team when observer available

Team discussed patient but did not see patient: 107

Team saw patient but observer did not enter room: 39

118 (26.3%) PCC team

330 (73.7%) Standard teams

448 patient-team interactions on rounds observed

PCC, patient-centered care

Supplemental Figure: Checklist


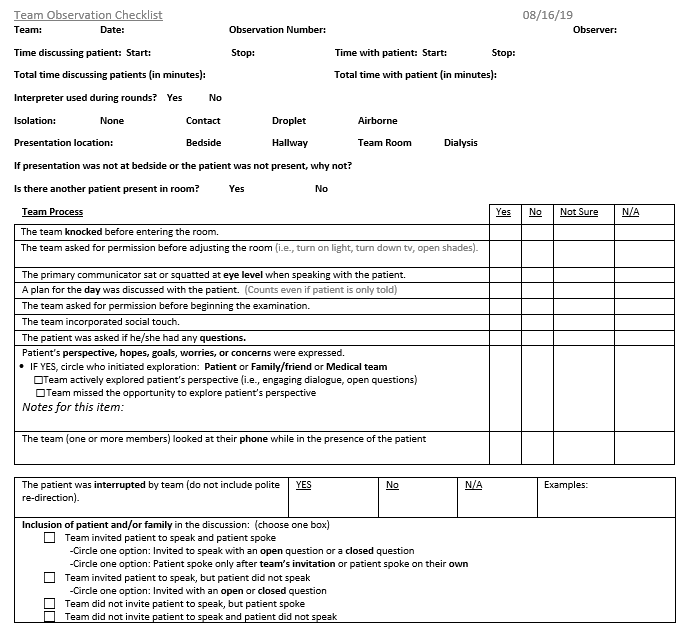

Supplement: Supplemental Material [file ZMEO_A_2024115_SM6347.docx]
